# Evaluation of current diagnostic methods for COVID-19

**DOI:** 10.1063/5.0021554

**Published:** 2020-12-01

**Authors:** Saadet Alpdagtas, Elif Ilhan, Ebru Uysal, Mustafa Sengor, Cem Bulent Ustundag, Oguzhan Gunduz

**Affiliations:** 1 Department of Biology, Van Yuzuncu Yil University, 65080 Van, Turkey; 2 Center for Nanotechnology and Biomaterials Application and Research (NBUAM), Marmara University, 34722 Istanbul, Turkey; 3 Department of Bioengineering, Faculty of Engineering, Marmara University, 34722 Istanbul, Turkey; 4 Department of Bioengineering, Faculty of Chemical and Metallurgical Engineering, Yildiz Technical University, 34210 Istanbul, Turkey; 5 Vocational School of Health Care Services, İstanbul Yeni Yuzyil University, 34010 Istanbul, Turkey; 6 Department of Metallurgical and Materials Engineering, Faculty of Technology, Marmara University, 34722 Istanbul, Turkey

## Abstract

Severe acute respiratory syndrome coronavirus 2 (SARS-CoV-2) is the agent responsible for the coronavirus disease of 2019 (COVID-19), which triggers lung failure, pneumonia, and multi-organ dysfunction. This enveloped, positive sense and single-stranded RNA virus can be transmitted through aerosol droplets, direct and indirect contacts. Thus, SARS-CoV-2 is highly contagious and has reached a pandemic level in a few months. Since COVID-19 has caused numerous human casualties and severe economic loss posing a global threat, the development of readily available, accurate, fast, and cost-effective diagnostic techniques in hospitals and in any places where humans spread the virus is urgently required. COVID-19 can be diagnosed by clinical findings and several laboratory tests. These tests may include virus isolation, nucleic acid-based molecular assays like real-time polymerase chain reactions, antigen or antibody-based immunological assays such as rapid immunochromatographic tests, enzyme-linked immunosorbent assays, immunofluorescence techniques, and indirect fluorescent antibody techniques, electrochemical sensors, etc. However, current methods should be developed by novel approaches for sensitive, specific, and accurate diagnosis of COVID-19 cases to control and prevent this outbreak. Thus, this review will cover an overview and comparison of multiple reports and commercially available kits that include molecular tests, immunoassays, and sensor-based diagnostic methods for diagnosis of COVID-19. The pros and cons of these methods and future perspectives will be thoroughly evaluated and discussed.

## INTRODUCTION

On 31 December 2019, 27 cases of a pneumonia of unknown etiology were detected in Wuhan City, China. All these patients had the same clinical symptoms as dry cough, fever, dyspnea, and bilateral lung infiltrates. It has been estimated that all the cases are linked to the Wuhan animal market, which includes various animals such as poultry, bats, marmots, and snakes.^1^ The disease was named coronavirus disease of 2019 (COVID-19) by the World Health Organization (WHO) on 7th January 2020.^2^ COVID-19 caused by SARS-CoV-2 primarily targets the human respiratory system. Previous coronaviral outbreaks (CoVs) were the severe acute respiratory syndrome (SARS)-CoV and the Middle East respiratory syndrome (MERS)–CoV, which pose a significant threat to humans.^3^ The genome sequence of SARS-CoV-2 showed similarities of 79.0% and 51.8% with SARS-CoV and MERS-CoV, respectively, and is 87.6% closely related to bat-induced SARS coronavirus.^4^ Therefore, it is predicted that SARS-CoV-2 was transmitted to humans from bats.^5^

Coronaviruses, which are single-stranded RNA viruses with a diameter of 80–220 nm, have a crown-like appearance under electron microscopy due to their surrounding glycoproteins.^6,7^ Coronaviruses are divided into four groups, including alphaCoV (α), betaCoV (β), deltaCoV (γ), and gammaCoV (δ). While α- and β-viruses can infect mammals, γ- and δ-viruses tend to infect birds.^8^ Previously, human-susceptible alphaCoVs displayed low pathogenicity and caused mild respiratory symptoms similar to the common cold, while SARS-CoV and MERS-CoV, among the other two known betaCoVs, caused serious and potentially fatal respiratory tract infections.^8^ As a member of beta coronaviruses, SARS-CoV-2 has a positive single-stranded RNA and enveloped structure.^9^ The viral RNA contains specific genes that encode proteins for viral replication in ORF1 downstream regions like all coronaviruses.^10^

Angiotensin-converting enzyme-2 (ACE2) is used by the coronaviruses as a receptor for entrance to the related cell. These receptors are commonly found not only in lung epithelial cells but are also located in small intestinal enterocyte cells, heart cells, and kidney endothelial cells.^11^ It is proved that SARS-CoV-2 binds to ACE2^12^ through its spike protein. After membrane fusion, viral RNA is released into the cytoplasm, and then RNA replication is initiated. Viral proteins synthesized through the host cell and the replicated viral RNAs are combined. Eventually, vesicles containing virion are fused with the plasma membrane and released out of the cell through exocytosis.^12–15^ Each virion remains to infect other cells until the immune defense takes over the task.

The investigations demonstrated that the virus could be easily transmitted from symptomatic or asymptomatic individuals. SARS-CoV-2 is transmitted through respiratory droplets from coughing, sneezing, and also direct contact. Therefore, it is quickly spread primarily among family members, healthcare professionals, and other close contacts.^16^ The reported clinical symptoms in 1099 COVID-19 cases are fever (88.7%), cough (67.8%), fatigue (38.1%), sputum production (33.4), shortness of breath (18.6%), sore throat (13.9%), and headache (13.6%). Some of the patients also manifested gastrointestinal symptoms such as diarrhea (3.8%) and vomiting (5.0%).^17^ The elderly and those with chronic diseases have developed rapidly acute respiratory distress syndrome, septic shock, and coagulation dysfunction, which has even lead to death.^18^

A definite treatment or specific vaccine for COVID-19 have not been observed yet. However, several approaches are currently used for treatment to prevent the outbreak. One of these methods is the utilization of antiviral drugs such as Chloroquine and Remdesivir to disrupt the viral mechanism.^19^ Another approach is to benefit from convalescent plasma. Convalescent plasma is obtained from COVID-19 patients who recovered from the disease.^20^ However, investigations are still performed to find out the exact treatment and vaccine for COVID-19.

For the diagnosis of COVID-19, real-time reverse transcription-polymerase chain reaction (RT-PCR), computed tomography (CT), and various laboratory tests are widely used.^21^ In this review article, diagnostic approaches for COVID-19 and diagnostic kits approved by the FDA will be discussed in detail. Comprehensive information will be presented about principles, challenges, advantages, and disadvantages of diagnostic approaches.

## TARGETS FOR COVID-19 DIAGNOSIS

As mentioned before, the diagnostic tests for SARS-CoV-2 can be performed by detection of the virus or the immune response against the viral agent. As a direct diagnosis, RT-PCR assays generally target one or more of the SARS-CoV-2 genes such as open reading frame1a/b (ORF1a/b), ORF1b-nuclear shuttle protein14 (ORF1b–nsp14), RNA-dependent RNA polymerase (RdRp), envelope (E), spike (S), or nucleocapsid (N) genes.^22–24^ Besides, another virus detection method is antigen-based immunoassays, and they should target the structural proteins of SARS-CoV-2, namely, viral antigens. Among them, the S protein is usually utilized for the diagnosis because it is the major transmembrane protein of the virus and highly immunogenic. Moreover, the S protein has an amino acid sequence variation among coronaviruses, enabling the specific diagnosis of the novel virus. Therefore, it is usually utilized as a target, albeit other proteins such as E and particularly N protein can be used as a marker for direct or indirect detection of the virus.^25,26^ The use of more than one of these antigens in the assays is essential for the sensitivity and specificity of the assay. Therefore, molecular assays or immunoassays prefer more than one specific SARS-CoV-2 target.^27^ On the other hand, to detect the immune response for SARS-CoV-2, antibody-based immunoassays can be carried out. Although total immunoglobulins can be used as a target for this assay, usually preferred molecules are IgM and IgG.^28^

**FIG. 1.**
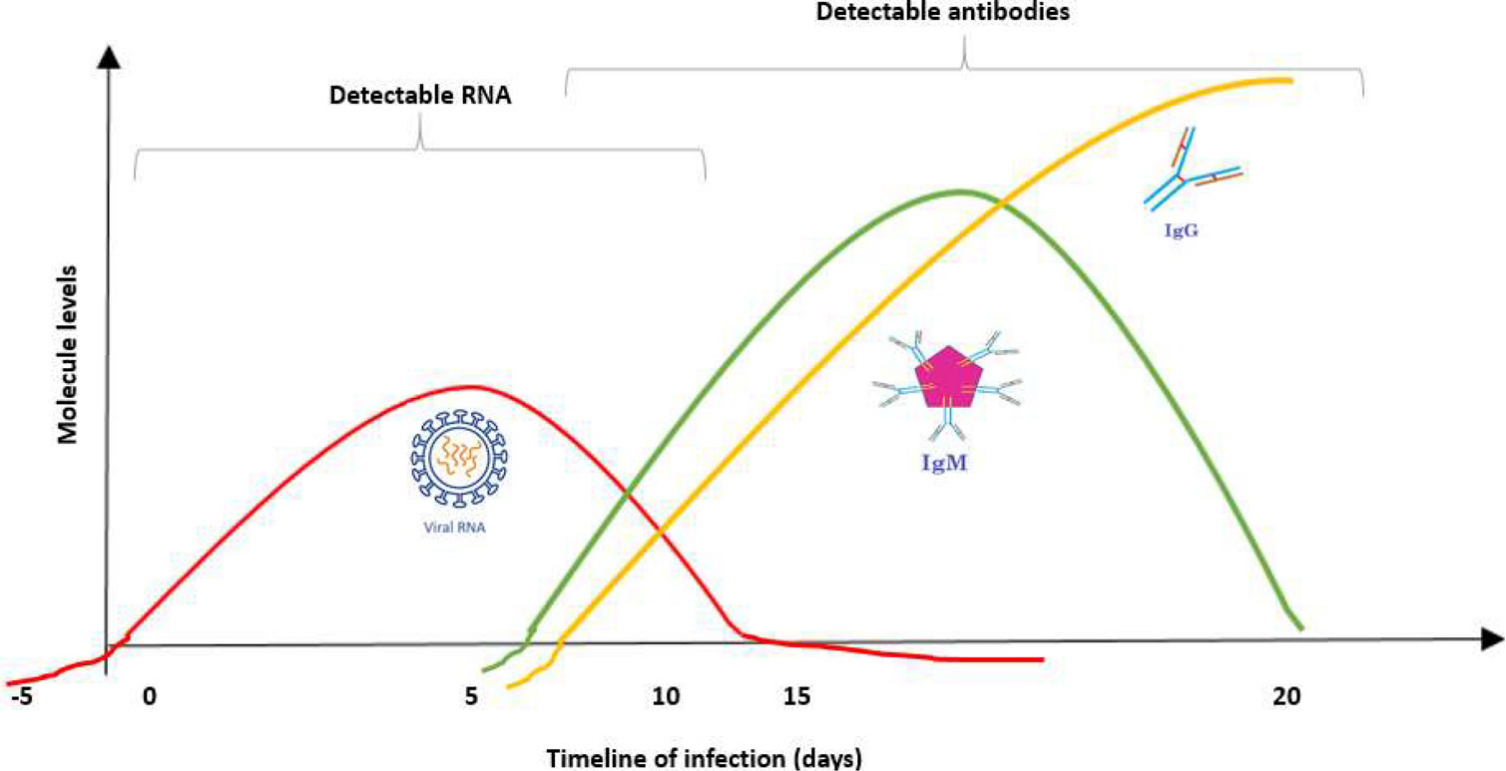
Diagnostic periods to detect SARS-CoV-2 infection and the immune response indicating previous exposure to SARS-CoV-2.

Viral antigens and antibodies (IgM and IgG) become detectable at different periods during infection (Fig. 1). The detection time of viral RNA, antigen, and antibodies depends on several parameters such as viral features, individual patient variability, and applied test. For these reasons, it is vital to select the appropriate diagnostic test within the correct timing for an accurate diagnosis.^24^ For instance, if you perform an immunoassay in the early stages of the infection when the antibodies cannot be produced yet, the test will be negative even though the disease is present. On the other hand, performing an RT-PCR test at the end of the disease or at the time that antibodies are produced will give a negative result due to the lack or low viral RNA load in the sample, respectively. Therefore, diagnostic periods and complementarity of the tests for the detection will affect the results in a negative manner.

## COMMON DIAGNOSTIC METHODS FOR RNA VIRUSES

**FIG. 2.**
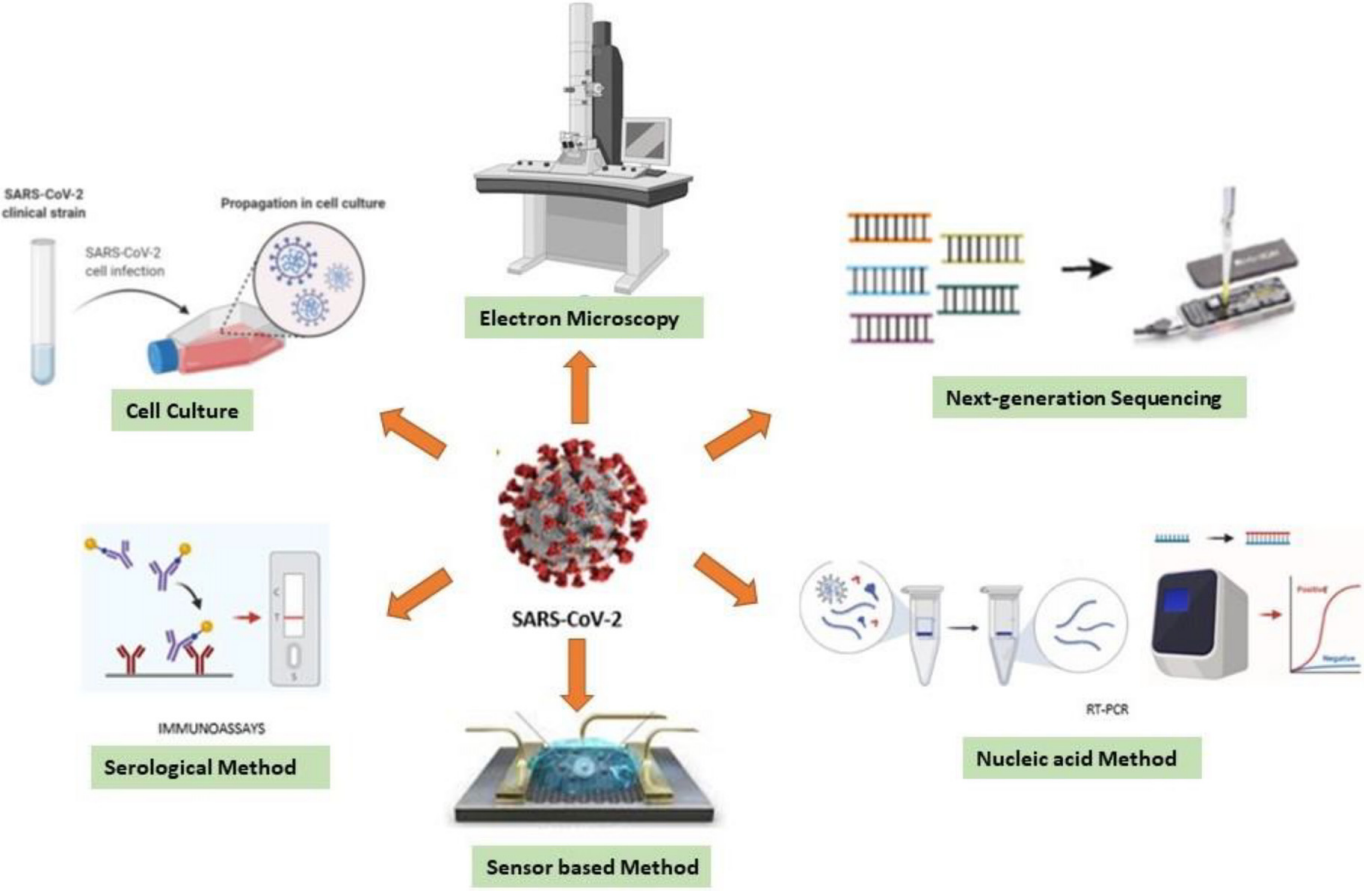
Diagnostic methods for RNA viruses.

Early and rapid detection of the virus provides both accurate and targeted therapy. Also, it reduces the consumption of nonspecific drugs, treatment costs, and morbidity.^29^ The diagnostic assays for RNA viruses are classified into five major categories: (i) cell culture, (ii) electron microscopy, (iii) next-generation sequencing methods, (iv) nucleic acid methods, and (v) serological methods (Fig. 2).

Cell culture, which is a traditional method, is utilized as a confirmation reference for most of the techniques that emerged for viral diagnosis. It is the only available technique for the detection and isolation of unknown viruses to characterize. However, the specificity issue and long incubation period have made the cell culture technique an undesirable method in urgent cases.^29,30^

The pioneering studies to identify pathogens are initiated with the imaging of the virus under an electron microscope (EM).^31^ The EM technique, which is still a vital diagnostic tool, is mostly used to eliminate inconsistencies of the results obtained from other methods. Two different EM techniques, immunoelectron microscopy (IEM) based on detecting a specific antibody-antigen complex and Solid-phase IEM (SPIEM) based on capturing viral particles directly on the solid surface of a grid, are used in the diagnosis of RNA viruses.^32,33^ Unfortunately, EM has some disadvantages such as low diagnostic sensitivity, the requirement of expensive equipment, and trained personnel.^34^

The next-generation gene sequencing method has become a technique that is frequently applied mainly in the epidemiology and characterization of pathogens.^30^ Although it is an accurate and reliable technique, its practical application is limited due to its high cost and expert requirement.

Among all diagnostic approaches, molecular methods and serology-based methods are usually preferred techniques for SARS-CoV-2 diagnosis. Mostly used molecular methods allow direct detection of the genetic material of the viruses from clinical samples such as blood, respiratory secretions, or body tissues.

## SIGNAL AMPLIFICATION TECHNIQUES

In infectious diseases, nucleic acids that belong to the pathogen are found in clinical samples in low copy numbers, and so amplification techniques should be used to detect the presence of pathogens.^35–37^ The amplification techniques are divided into three groups according to the applied principle. These techniques are based on (i) amplifying target nucleic acids, (ii) amplifying probes that bind to the target nucleic acids, and (iii) amplifying signals generated from target nucleic acids.^38^ The latter, signal amplification technique, is divided into three subgroups as branched DNA techniques, hybrid capture, tyramide signal amplification, and cleavage-based signal amplification.^39^

The branched DNA technique is based on the signal measurement of target nucleic acid after immobilization and then hybridization with multiple branched and labeled probes. The advantages of the technique are quantification capability, low contamination issue, applicability without a thermal cycler, or enzyme. In contrast, the technique has a long turnaround time and less sensitivity.^35,39,40^

In the tyramide signal amplification technique, the target nucleic acid is hybridized with a biotinylated probe and a nucleic acid-biotinylated probe complex (NBPC) is obtained. Hydrogen peroxidase, which carries streptavidin, is added to the medium and bound to NBPC. When inactivated tyramide is added to the medium as a substrate, the reaction results in the activated tyramide and precipitates in the hybridization site. The amount of precipitate indicates signal amplification. The technique is not preferred due to the low detection threshold.^41^

In the hybrid capture technique, hybridization is achieved with the complementary RNA probe of single-stranded target DNA (if target nucleic acid is RNA, the probe will be single-stranded DNA). The resulting DNA:RNA hybrid is transferred to the polyclonal anti-DNA:RNA hybrid antibody-coated medium and labeled with the conjugated enzyme-labeled monoclonal antibody. Signal amplification reveals when the enzyme interacts with the chemiluminescence substrate.^42^ The total duration for the assay is about 3 h, and the test provides a theoretically 3000 times amplification.^35,40,43^

The cleavage-based signal amplification technique is much stronger than the hybrid capture technique.^43^ The technique is based on the principle of hybridization of two target-specific oligonucleotides (probes) with DNA, enzyme digestion, and hybridization of the product with the FRET cassette and measuring the fluorescence generated with the enzyme digestion.^35^ There is not any product developed using this method yet for the detection of SARS-CoV-2.

Swift and Arbor Bioscience companies have developed a kit for SARS-CoV-2 sequencing by combining the hybrid capture technique with the next-generation sequencing method. In another next-generation sequencing kit developed by BioCat, hybridization is achieved with biotinylated RNA probes, and the hybrid product is held with streptavidin-coated magnetic beads and amplified with PCR.

## ELECTROCHEMICAL-BASED SENSOR SYSTEMS

In the case of viral infections, the development of portable DNA sensors is essential in medicine. It is based on the principle that the genetic material of the virus (RNA or cDNA) can be detected by using a complementary probe.^44–46^ Several transduction principles are used in the development of such DNA sensors, among which electrochemical methods with their unique advantages have been explored, in particular, in terms of sensitivity, the limit of detection, and susceptibility to miniaturization.^47–50^

Unlabeled electrochemical detection of DNA hybridization is presented as a potential approach for the diagnosis of COVID-19 by Tripathy and Singh.^51^ In this reported approach, the target nucleotide can be SARS-CoV-2 specific viral RNA or the corresponding cDNA or any unique sequence specific to them. A complementary single strand probe with thiol modification at one end can be designed to this target sequence, and the thiol-modified probe can be connected to the gold sensing electrodes through the gold-thiol self-assembly. As a result, it is made ready for diagnosis by blocking nonspecific binding sites on the sensing surface, and the target nucleotide hybridizes with a complementary probe under appropriate physiological conditions when applied to the sensors. This hybridization can be recorded using electrochemical techniques, thereby developing a diagnostic scheme.

For the detection of SARS-CoV-2, Seo *et al.* have developed a field-effect transistor-based biosensor. This sensor was produced with coated graphene leaves of FET with specific antibodies targeting the SARS-CoV-2 spike protein. The device has the sensitivity to detect SARS-COV-2 at the femtogram level in milliliters and does not require sample preparation. This sensor is a high-precision immunological diagnostic device developed for COVID-19.^26^

## MOLECULAR METHODS

The development of molecular diagnostic techniques to detect SARS-CoV-2 depends on understanding the proteomic and genomic composition of the virus. Nucleic acid amplification tests currently available for diagnosis of COVID-19 include RT-PCR and reverse transcription loop-mediated isothermal amplification (RT-LAMP).^52,53^

Three regions conserved among SARS-related viral genomes were discovered: (i) the E gene (envelope protein gene), (ii) the RdRP gene (RNA-dependent RNA polymerase gene) in the open reading frame ORF1ab region, and (iii) the N gene (nucleocapsid protein gene).^54^ The U.S. Food and Drug Administration (FDA) concluded that a negative RT-PCR test result does not entirely exclude SARS-CoV-2 infection and would not be used as a single reference for diagnosis.^55^

For SARS-CoV-2, transferring the molecular diagnostic tests from the laboratory to the point of care (POC) applications is crucial for increasing the testing capacity, also potentially reducing the assay duration, and supporting early identification of positive cases.^56,57^ Numerous points of care molecular tests have received Conformité Européenne (CE) mark or Food and Drug Administration (FDA) approval. The point of care molecular testing uses the same basic technology as laboratory-based testing but automates various steps. MicrosensDx RapiPrep © COVID-19 and Abbott ID NOW COVID-19 tests are based on isothermal nucleic acid amplification techniques, while Credo VitaPCR COVID-19 assay, Cepheid Xpert SARS-CoV-2, MesaBioTech Accula SARS CoV-2, GenMark ePlex SARS-CoV-2, and tests are PCR based. Also, a Spartan Cube CYP2C19 System is developed in Canada, which is based on PCR.^58^

Loop-mediated isothermal amplification (LAMP) has been developed as a fast, accurate, reliable, and cheaper technique to amplify the target region at a single reaction temperature instead of the thermal cycle required in RT-PCR.^59^ The advantage of the LAMP method to RT-PCR is that the amount of DNA produced is much higher, and a positive test result can be viewed visually without the need for an additional analysis step. While two studies reported that RT-LAMP methods showed more than 97% sensitivity in targeting the ORF1ab gene compared to RT-PCR, another study showed that both methods had the same sensitivity and both were able to detect a 20-fold diluted sample.^60–62^ It also showed that the technique was highly specific, as six to eight primers were used in the RT-LAMP analysis to identify eight different regions on the target DNA.^62,63^

Another molecular method used to detect SARS-CoV-2 is clustered regularly interspaced short palindromic repeats (CRISPR)-based analysis. Two known companies developed this method. These are Mammoth Biosciences and Sherlock Biosciences. The SHERLOCK method developed by Sherlock Biosciences uses Cas13, which can cut reporter RNA sequences after activation by the SARS-CoV-2-specific guideline RNA.^64^ The DETECTR test developed by Mammoth Biosciences relies on the cut of the reporter RNA by Cas12a to accurately detect viral RNA sequences of the E and N genes, followed by isothermal amplification of the target, causing a visual reading with a fluorophore.^65^

Microarray assays are based on the formation of cDNA from viral RNA by reverse transcription and subsequent labeling of the cDNA with specific probes, which have been used for rapid high-throughput detection of SARS-CoV nucleic acids. Labeled cDNAs are loaded into the wells of microarray trays containing solid phase oligonucleotides on their surface. If hybridization occurs, the cDNAs remaining into the well indicate the presence of virus-specific nucleic acid.^66^ Microarray analysis has proven to be useful in identifying SARS-CoV-related mutations and has been found to detect 24 single nucleotide polymorphisms (SNPs) associated with mutations in the SARS-CoV spike (S) gene with 100% accuracy.^67^ The amplicon-based metagenomic sequencing technique for identifying SARS-CoV-2 is based on a dual approach that involves both metagenomic sequencing and the use of amplicon-based sequences. Metagenomic sequencing can quickly identify SARS-CoV-2 virus and other pathogens that contribute to secondary infections that affect the severity of COVID-19 symptoms by providing analysis of the background microbiome of infected individuals. Amplicon and metagenomic MinION-based sequencing were used to rapidly sequence the SARS-CoV-2 genome and other microbiomes in nasopharyngeal swabs obtained from COVID-19 patients.^68^ Using sequencing methods, it is provided to detect mutations in the SARS-CoV-2 that may occur over time, to identify variations in different sites of the world, and to offer new targets for diagnosis and treatment.

Microarray analysis is the most prominent method; microarray analysis has confirmed that it has 100% accuracy for identifying and detecting SARS-CoV-related mutations. Identifying and detecting mutations can lead further studies that can offer new types of diagnosis and treatments. On the other hand, all over the world, RT-PCR is used, but it is not sufficient as a single reference. In comparison to RT-PCR, RT-LAMP, which has no additional analysis step, would be more prominent.

## IMMUNOLOGICAL ASSAYS

Although the molecular techniques are effective and sensitive for COVID-19 diagnosis, they suffer from numerous limitations. Sampling failures/quality, complicated protocols, long turnaround times, the dependency on certified laboratories, expensive equipment, and trained people are the most known drawbacks of this conventional method. Moreover, false-negative results of these tests triggered to develop other supportive tests for accurate diagnosis of COVID-19 at this pandemic stage to prevent the spread of viruses.^27,28,69,70^ As shown in Fig. 1, the sensitivity of a nucleic acid test to be performed in the earlier period of the disease or the recovery stage will be very low and the test will give false negative results even though in the presence of infection. Particularly, the specimens collected in the late stage of disease may not have enough viral load and lead to a low positive rate for RT-PCR. Albeit, the same clinical samples also have high amounts of virus-specific antibodies. Therefore, to detect the antibodies by immunoassays is a preferable method at this stage.^27^ To overcome all the above-mentioned limitations of molecular tests, immunoassays can be used as a complementary method due to their advantages such as comparatively easier sampling/performing, less requirement for technical expertise, and equipment. On the other hand, immunoassays can also be designed not only for antibody detection but also for antigen detection as an alternative for RT-PCR.^24,28^

Another issue about molecular tests is reporting very mild cases of infection or asymptomatically infected cases. According to the studies, the symptoms of COVID-19 differ among individuals, ranging from asymptomatic infection to severe cases.^71,72^ Asymptomatic carriers are defined as individuals who are positive for viral RNA but without any symptoms during the screening of close contacts. It is also challenging to identify and quarantine them by RT-PCR.^23,73^ Confirming these suspected COVID-19 cases as early as possible with the help of serological testing can reduce exposure risk. That is, the combination of nucleic acid assay and immunoassay is a more sensitive and accurate approach for the diagnosis of COVID‐19.^27,74^

Immunoassays are available in a broad range of different types but mainly consist of an antibody or antigen immobilized on a matrix, which binds viral targets or antibodies in clinical samples (respiratory samples or blood derivatives). It is then possible to detect a virus-specific immune signal to confirm the presence of the antigen or antibody by adding a further reporter protein.^75,76^ That is, immunoassays can detect viral antigen (antigen tests) or immune response (antibody tests), respectively.

## ANTIBODY-BASED ASSAYS

After the viral infection, the body protects itself via immune defense and produces specific antibodies for this pathogenic organism. Antibody assays can be used to detect this immune response, and also, the previous exposure to SARS-CoV-2 can be determined in this way. The body typically takes a certain period to initiate a response to the infection. Therefore, the utility of antibody assays to diagnose acute infections in the early stages is limited. Because during this period, the body is not yet familiar with the related antigen and it will take time to recognize it and develop an appropriate immune response. Therefore, an antibody test to be applied at this stage will give a false negative result due to the absence of antibodies despite the presence of the disease. During the primary reaction of a virus, IgM antibodies are the first to appear. However, they are relatively short-lived and disappear after a few weeks. The detection of these antibodies implies potentially active or recent infection. On the other hand, IgG is the major antibody of the immune defense and provides long-lasting immunity against the same virus for re-infection.^24,77^ Thus, detection of both IgM and IgG can give information about the virus infection time course. The kinetic specificities of serum-specific antibody production after the SARS-CoV-2 infection are still under investigation.^69,78^ According to the acquired data, there is a decrease in levels of immunoglobulins in COVID-19. This indicates the effects of the disease on antibody-producing B lymphocytes. Even though viral antigens have shown potential for triggering antibody production, lymphopenia may have caused the depletion of immunoglobulins.^79^ Although the antibody amount is reduced, IgM and IgG play critical roles in the immunity of COVID-19. There are some reports on this issue.^74,80^ In one of them, Long *et al.* studied for the acute antibody responses to SARS-CoV-2 with 285 patients by using a magnetic chemiluminescence enzyme immunoassay (EIA).^74^ They had observed three types of seroconversion; synchronous seroconversion of IgG and IgM, IgM seroconversion earlier than that of IgG, and IgM seroconversion later than that of IgG. That is, seroconversion for IgG and IgM occurred simultaneously or sequentially. Both IgG and IgM quantities plateaued within 6 days after seroconversion. Within 19 days after the symptom onset, 100% of patients assayed positive for IgG. As it is known, the production of antibodies during an acute phase infection is consistent in most patients; albeit, it may be delayed, weak, or ineffective in the elderly members and in those who have immunosuppression or other treatments that weaken the immune response, such as chemotherapy. Except for the immunodeficiency situation, the presence of a specific antibody can be detected, avoiding false‐negative.^24,71^ Thus, to increase the sensitivity of COVID‐19 diagnoses, antibody-based immunoassays can be utilized for the detection of IgM and IgG. A positive antibody test indicates current or recovered infection; however, negative test results do not exclude COVID-19 disease. In summary, viral RNA detection assays (RT-PCR, next-generation sequencing, etc.) and tests to detect antibodies should not be considered competing alternatives. Both assays are clinically relevant and complementary but must be utilized at different time points during the clinical course of the disease, taking consideration of their relevant diagnostic perspectives.^24,27^

However, the specificity of the antibody assays should be considered and if required retested because these tests can cross-react with antibodies produced against other coronaviruses, which are prevalent in the general population. As it is known, whole-genome sequencing of this novel virus has shown that it has a high degree of nucleotide identity with SARS-CoV.^22^ Thus, any antibody tests used to detect SARS-CoV-2 requires the identification of and ruling out cross-reactivity with common coronavirus strains.^81^

For high-throughput screening, a protein chip or microarray technologies can be used as an immunoassay for diagnosis. As a general protocol, the clinical sample is incubated on the chip. If antibodies produced against SARS-CoV-2 are present in the clinical sample, an interaction between the viral antigen and antibody is detected.^82^ Proteome microarrays for the diagnosis of SARS-CoV-2 are currently in development, and by these tests, it will be possible to identify, profile, and compare antibody responses in sera samples to inform vaccine development or screen viral antigens to find and characterize appropriate immunodominant epitopes for *in vitro* diagnostics research.^83,84^

Consequently, the combination of both tests is the optimal method for considering the whole stages of the disease and determining SARS-CoV-2 infection. This result had also been confirmed by Rashid *et al.*, and they had reported that when IgM Enzyme Linked Immunosorbent Assay (ELISA) is combined with PCR, the positive detection rate is remarkably increased (98.6%) for each patient compared to a single qPCR test (51.9%).^27,80^

## ANTIGEN-BASED ASSAYS

A key aspect of limiting this pandemic outbreak is to ensure the early and accurate diagnosis of the disease and provide appropriate quarantine conditions for those symptomatic or asymptomatic patients.^80^ As it is known, antibody detection assays have drawbacks for early case detection due to the absence of antibodies at the first stage of the disease. Therefore, at this stage, the viral RNA or viral antigens should be used for diagnosis via RT-PCR or antigen-based immunoassays, respectively. For an acceptable diagnosis, antibody-based immunoassays should be supported by one of these methods. Due to their ease-of-use and turnaround time, antigen tests can be preferred for this issue, if they are sufficiently sensitive. These tests incorporate the antibodies that are specific to viral antigens and can determine the presence of viral immunogen as a precursor of infection. They are fast and also of low cost relative to molecular assays; albeit, they are generally less sensitive.^85^ If proven to have relatively high specificity and sensitivity, antigen detection tests may be of value in the early diagnosis of COVID-19 but cannot be used for past exposure.^24^

These immunoassays can be performed with various clinical samples due to their variable load of antigen or antibody. While whole blood, serum, or plasma can be used as a specimen for antibody-based immunoassays, the upper or lower respiratory samples are used for antigen-based immunoassays. To acquire blood samples are easier and risk-free compared to respiratory samples. For safe and accurate sampling for diagnosis, different clinical samples such as stool, urine, and saliva are under investigation to screen their viral load and diagnostic potential for COVID-19.^28,86,87^

Immunoassay systems have different turnaround times, specificity, and sensitivity. These parameters are generally related to the binding assay type. There are numerous special binding assays such as immunofluorescence assays (IFAs), rapid immunochromatographic assays, chemiluminescence immunoassays (CLIA), and enzyme-linked immunosorbent assays. Several researchers had utilized each of these binding principles for their diagnostic assays and reported some concluding remarks about the detection time, specificity, and sensitivity of their immunoassays.^24,77,83,88,89^ For instance, Zhang *et al.* used an automated chemiluminescence immunoassay to evaluate the value of immunoassays for diagnosis of COVID-19 and the antibody production process after infection.^77^ This commercial immunoassay kit including magnetic particle-coated S and N protein was used for antibody detection using an automatic chemiluminescence immunoassay analyzer in 30 min. Serum IgM and IgG antibodies against SARS-CoV-2 were measured in 736 participants. According to their investigation, the course and speed of antibody production are correlated with disease severity and have diversity in different individuals. Nucleic acid tests should be supported by such tests. In another study, Loeffelholz and Tang developed an accurate, rapid, and simple, immunochromatographic fluorescence assay for detecting N protein of SARS-CoV-2 in nasopharyngeal swab and urine samples for diagnosis of COVID-19 within 10 min.^86^ The investigation was performed with 239 participants with suspected SARS-CoV-2 infection. The viral loads were also checked using nucleic acid tests, and results were used as the reference standard for immunoassay. The tests gave positive results with urine and nasal samples, and also, the earliest participant after 3 days of fever was able to be identified using this method. These findings indicate that nucleocapsid protein assay is an accurate, rapid, early, and simple method for diagnosis of COVID-19. On the other hand, Li *et al.* have developed a rapid and simple point‐of‐care lateral flow immunoassay that can detect IgM and IgG antibodies simultaneously against the RBD domain of SARS‐CoV‐2 spike protein in human blood within 15 min.^69^ Its clinical efficacy has been validated. The sensitivity/specificity of this test has been measured using blood samples collected from 397 PCR, which confirmed COVID‐19 patients and 128 negative patients at eight different clinical sites. The observed testing sensitivity and specificity have been reported to be 88.66% and 90.63%, respectively. In addition, different types of venous and fingerstick blood samples have been evaluated and compared for diagnosis, and the results displayed a detection consistency among samples. These researchers claimed that the IgM–IgG combined assay has better utility and sensitivity compared to a single IgM or IgG test. They also propose this test for the rapid screening of SARS‐CoV‐2 carriers, symptomatic or asymptomatic, in hospitals, clinics, and test laboratories. Still, there are relatively few reports on COVID‐19 patient diagnoses through serological tests. To fill this gap and to increase the sensitivity of COVID-19 diagnoses, Xie *et al.* used both commercial IgM–IgG immunoassay and nucleic acid assay for detection and tested this combination with 56 patients with suspected SARS‐CoV‐2 infection.^27^ In all patients, IgG and IgM antibodies against the SARS‐CoV‐2 E and N protein in serum samples were measured using chemiluminescence immunoassay, and the gene encoding nucleocapsid protein and ORF1ab were amplified by RT-PCR. They realized that despite negative nucleic acid test results, all patients showed high specific IgG concentrations, suggesting SARS‐CoV‐2 infection. Based on this study, they suggested that such a combination would be a more sensitive and accurate approach for diagnosis and early treatment of COVID‐19. As it is known, the turnaround time is as significant as accuracy and also, diagnosis at early stages is crucial. To provide these advantages, Sona Nanotech (Halifax, Canada) tried to develop a quick-response lateral-flow test prototype to screen the SARS-CoV-2 virus in clinical samples to produce results in 5–15 min.^90^ It offers a unique advantage over other lateral flow tests as it detects the presence of the COVID-19 virus. To date, only the competitive lateral flow tests that have been announced for sale are serological assay tests, which are designed to identify IgM and IgG antibodies that present post-infection. Therefore, to detect the viral particle with the immunoassay is a superiority for this investigation. As a complementary method for RT-PCR, Zhong *et al.* developed ELISA and chemiluminescence methods to detect IgM and IgG antibodies in serum samples and both assays are investigated in the presence of S or N proteins, respectively. These tests were performed with 47 COVID-19 positive patients and 300 healthy participants, and results were compared with those obtained from the nucleic acid detection assay. The research indicated that ELISA and chemiluminescence methods to detect IgG and IgM antibodies by the recombinant N and S proteins of SARS-CoV-2 were more consistent with the nucleic acid detection assay. According to the given data, the S-based IgM ELISA was more sensitive than the N-based IgM ELISA.^91^ This result can be explained by the immunogenicity of S1 protein, which may easily stimulate the body to produce the IgM antibody, especially during early infection.^92^

## COMPARISON OF US FDA CERTIFIED COMMERCIAL KITS FOR COVID-19

According to FIND data (Foundation for Innovative New Diagnostics), numerous diagnostic approaches have been developed to determine either SARS CoV-2 or the immune response (IgM or/and IgG) against the virus.

These assays include a range of laboratory-based tests and rapid tests designed for near-patient testing to accelerate clinical diagnosis and increase testing quantity; however, a majority of these tests are yet to be validated for utilization in clinical settings.^24^ At present, there are 333 molecular tests that are developed or in the development stage as a diagnostic tool for COVID-19, but only 31 of them had been commercialized with US FDA approval. All these tests targeting one or more regions of the viral RNA require automated lab systems. Also, 347 immunoassays (developed or in the development stage) are known for the diagnosis of COVID-19. But only 12 of them (11 of them are antibody-based and only one of them is antigen-based) had been approved by the US FDA. All these FDA approved immunoassays and only some of the representatives of molecular assays are provided together with their features, principles, and analytical performances in Table I.

**TABLE I.** US FDA-approved commercial diagnostic kits for COVID-19 diagnosis. LFI: Lateral Flow Immunoassay; EIA: Enzyme Immunoassay; IFA: Immunofluorescence Assay; ECLIA: Electrochemiluminescence Immunoassay; ELISA: Enzyme Linked Immunosorbent Assay; CLIA: Chemiluminescence Immunoassay; N: Nucleocapsid; S (1,2): Spike (1,2); E: Envelope; RBD: Receptor Binding Domain; ORF: Open Reading Frame; and RdRp: RNA-dependent RNA polymerase.

Molecular-based assays^a^								
Number	Manufacturer	Kit name	Detection target	Duration (min)	Specimen type	Assay	Sensitivity (%)	Specificity (%)
1	Altona Diagnostics	RealStar^®^ SARSCoV-2 RT-PCR Kit 1.0	E and S genes	∼90^b^	Nasal, nasopharyngeal, and oropharyngeal swabs	RT-PCR (BioRad CFX96 deep well)	92	100
2	Atila BioSystems, Inc.	Atila iAMP COVID-19 Detection (isothermal detection)	ORF1ab and N genes	∼60	Nasal, nasopharyngeal, and/or oropharyngeal swabs	RT-PCR (BioRad CFX96 deep well)	100	99 (ORF1ab)^c^ 100 (N)
3	BGI Health (HK) Co., Ltd.	Real-time fluorescent RT-PCR kit for detection 2019-nCOV (CE-IVD)	ORF1 gene	180	Throat swab and Bronchoalveolar Lavage Fluid	Fluorescent RT-PCR (Roche LightCycler 480)	100	99^c^
4	Primerdesign, Ltd.	Coronavirus COVID-19 genesig^®^ Real-Time PCR assay	RdRp gene	120	Nasopharyngeal, oropharyngeal swabs, and sputum	Fluorescent RT-PCR (LightCycler 480)	100	100
5	SD Biosensor Inc.	Standard M nCoV Real-Time Detection Kit	E and ORF1 genes	∼90	Nasopharyngeal swabs and throat swab	Fluorescent RT-PCR (Roche LightCycler 480)	100	97 (E)^c^ 99 (ORF1)^c^
6	Seegene, Inc.	Allplex™ 2019-nCoV assay	E, N and RdRp genes	∼110 (After extraction)	Sputum, Throat swab, Nasopharyngeal, Bronchoalveolar lavage	RT-PCR (BioRad CFX96)	100	100

Antigen-based manual or automated immunoassays								
Number	Manufacturer	Kit name	Detection target	Duration (min)	Specimen type	Assay	Sensitivity (%)	Specificity (%)
1	Quidel	Sofia 2 SARS Antigen FIA	N protein	15–20	NasalNasopharyngeal samples	IFA	80	100

Antibody-based manual or automated immunoassays								
**1**	Bio-Rad Laboratories, Inc.	Platelia SARS-CoV-2 Total Ab	IgG, IgA, IgM (against N protein)	∼120	SerumPlasma	EIA	97.50	99.56
**2**	Calbiotech, Inc.	ErbaLisa COVID-19 IgG ELISA	IgG (against S protein)	∼ 60	Serum	ELISA	98.30	98.10
3	EUROIMMUN AG	Anti-SARS-CoV-2 ELISA (IgG)	IgG (against S1 RBD)	120	SerumPlasmaWhole blood	ELISA	94.40	99.60
4	Ortho Clinical Diagnostics	VITROS^®^ Immunodiagnostic Products Anti-SARS-CoV-2 IgG	IgG (against S protein)	∼85	SerumPlasma	CLIA	83.30	100
5	Ortho Clinical Diagnostics	VITROS^®^ Immunodiagnostic Products Anti-SARS-CoV-2 Total	IgG, IgA, IgM (against S1 protein)	∼90	SerumPlasma	CLIA	80	100
**6**	Roche	Elecsys Anti SARS CoV-2	IgG (against N protein)	18	SerumPlasma	ECLIA	88.1	99.81

Antibody-based rapid diagnostic kits								
Number	Manufacturer	Kit name	Detection target	Duration (min)	Specimen type	Assay	Sensitivity (%)	Specificity (%)
1	Cellex, Inc.	Cellex qSARS-CoV-2 IgGIgM Cassette Rapid Test(Colloidal Gold)	IgM/IgG (against N & S protein)	15–20	SerumPlasmaWhole blood	LFI	93.80	95.60
2	Jiangsu Superbio Biomedical Technology (Nanjing) Co., Ltd	SARS-CoV-2 (COVID-19) IgM/IgG Antibody Fast Detection Kit (Colloidal Gold)	IgM/IgG (Against S RBD)	15	SerumPlasmaWhole blood	LFI	88.66	90.63
3	ScheBo Biotech AG	ScheBo SARS-CoV-2 Quick	IgM/IgG	15	SerumPlasmaWhole blood	LFI	97.5	99.5 (IgM) 100 (IgG)
4	Autobio Diagnostics Co., Ltd.	Anti-SARS-CoV-2	IgM/IgG	<15	SerumPlasmaWhole blood	LFI	95.7 (IgM) 99 (IgG)	99.5

^a^ https://www.finddx.org/covid19/pipeline/?avance=Commercialized&type=all&test_target=all&status=US+FDA&section=molecular-assays&action=default#diag_tab (visit the website for other 25 tests which are approved by the US FDA molecular test). Updated information on evaluation of the diagnostic tests can be found in https://www.finddx.org/covid-19/sarscov2-eval/.

^b^ Reaction period in the thermal cycler.

^c^ Further investigation needed to determine if apparent false positives are truly false positives or whether they are due to a false negative reference standard result.

The selection of the most appropriate assay will depend on the availability of resources and the local epidemiological situation. The ASSURED (Affordable, Sensitive, Specific, User-friendly, Rapid and robust, Equipment-free, and Deliverable to end-users) criteria proposed by the WHO can be used as a guide to select the most appropriate diagnostic assay among numerous available alternatives.^93^ Among all these tests, particularly rapid tests are attractive in such pandemic situations because rapid assays can be applied in remote and low-income regions where molecular assays or automated immunoassays cannot be utilized.^80^ Many of these rapid tests that are available or in development for the detection of SARS-CoV-2 are based on antigen and antibody immunoassays. The majority of them are based on lateral flow assays, and cellulose-based devices intended to detect the target analyte in a liquid sample. These qualitative or semi-quantitative *in vitro* diagnostic medical devices can be used singly or in a small series.^24^ According to FIND verification, only four rapid antibody-based tests are approved by the US FDA (Table I).

## FUTURE PERSPECTIVES AND BARRIERS

Currently, nucleic acid-based molecular tests are still considered the gold standard for the diagnosis of COVID-19 disease. The detection time has been reduced to 30 min. It is a highly sensitive specific technique in the diagnosis of infected COVID-19 patients. RT-PCR is the most used test among molecular techniques. The requirement of reliable control for confirmation, the necessity for expensive equipment and trained person, certificated reagents, and laboratory facilities are known disadvantages of molecular methods. In future applications, alternatives such as LAMP and CRISPR-Cas methods may become more common as they are a less costly, simple procedure.^29,94^ Serological methods such as antibody or antigen-based diagnostic tests may be considered more desirable in the future because of their cycle times and point-of-care (POC) applicability, if their accuracy and reliability can be improved over molecular techniques. It is necessary to identify the disease and assess the sensitivity and specificity of the tests, especially during the acute phase of COVID-19 infection. However, in combating a worldwide pandemic, serological methods and data will become increasingly important to understand the history of pandemics and predict the future.^89,95^ As one of the next-generation techniques, whole-genome sequencing is the most promising method for COVID-19 characterization, genomic surveillance, understanding viral transmission, and pathogenicity, identifying viral mutations, and developing therapy. However, this costly and time-consuming method limits its practical application. Electrochemical sensors, one of the new generation methods, are thought to be more sophisticated in the future for the selective and sensitive detection, identification, and quantification of viruses. Biosensor-based virus detection systems that utilize nanotechnology and microfluidics and instrumental advances are predicted to be among the most promising technologies in pandemic situations like COVID-19.^96^ In the future, it is thought that easier and more mature biosensor platforms will replace RT-PCR. Further studies are needed to compare existing methods in terms of robustness, reproducibility, reliability, and sensitivity.^97^

To summarize, current analysis methods are not sufficient to distinguish infected persons, especially in public places. There is a need to produce POC devices that can detect infections on the site without the need for professionally trained personnel. In future applications, POC diagnostic devices and tests are increasing in popularity, especially in the case of a worldwide pandemic such as COVID-19.^97,98^

## CONCLUSION

Early diagnosis is essential to identify cases and prevent infection in such pandemic outbreaks. In the current scenario, various technologies are available to provide better diagnostic capabilities to the community. For accurate and precise diagnosis, correct sampling and sampling periods are significant. In current COVID-19 cases, RT-PCR testing is the gold standard for the etiological diagnosis of the virus. On the other hand, antibody-based immunological tests are practical and easy-to-use methods for rapid screening of a whole society and verification of the molecular analysis. Rapid diagnosis kits are in demand for providing rapid diagnosis especially in emergencies, at the bedside and in several places. Even if there are various doubts about its accuracy and sensitivity, it is an indispensable, important method for early diagnosis in the case of an existing pandemic. Nowadays, immunological tests and RT-PCR applications are critical diagnostic systems used to combat the COVID-19 outbreak that affects our life and the global economy. In summary, the use of the current tests can be done alone or in combination; although it can detect COVID-19 cases, there is still an urgent need to develop more practical, precise, and accurate detection tests that show results faster for an enhanced quality of life.

## AUTHORS' CONTRIBUTIONS

O.G., C.B.U., M.S., and S.A. conceived the review topic and outlined the manuscript draft. E.I. drafted the general information and future perspective/barrier sections, E.U. drafted molecular- and sensor-based methods, S.A. drafted the immunoassays and also compared the FDA approved commercial kits. All the authors edited, revised the draft critically, and approved the final version of this manuscript.

## Data Availability

Data sharing is not applicable to this article as no new data were created or analyzed in this study.
